# Preliminary study of the effect of low-intensity focused ultrasound on postpartum uterine involution and breast pain in puerperal women: a randomised controlled trial

**DOI:** 10.1038/s41598-024-51328-9

**Published:** 2024-01-05

**Authors:** Dongmei Wei, Jun Yue, Jian Meng, Jing Gao, Lei Yang, Xiaoyu Niu, Zhijian Wang

**Affiliations:** 1 Department of Gynecology and Obstetrics, West China Second Hospital, Sichuan University, Chengdu, 610041 China; 2 Key Laboratory of Birth Defects and Related Diseases of Women and Children (Sichuan University), Ministry of Education, Chengdu, China; 3 Department of Gynecology and Obstetrics, Sichuan Provincial People’s Hospital, Chengdu, China; 4 Department of Medicine, LIFU Research Institute, Chengdu, China; 5 Department of Gynecology and Obstetrics, Southern Hospital, Southern Medical University, Guangzhou, China

**Keywords:** Urogenital diseases, Outcomes research, Health care, Medical research

## Abstract

To evaluate the safety and efficacy of low-intensity focused ultrasound (LIFU) therapy in facilitating fundus descent and relieving postpartum breast pain compared with sham treatment. A multicentre, randomised, sham-controlled, blinded trial was conducted. A cohort of 176 eligible participants, who had normal prenatal check-ups and met the inclusion and exclusion criteria, were recruited from three medical centres and subsequently randomized into either the LIFU or sham group. All participants received three treatment sessions, wherein LIFU signal was applied to the uterus and breast sites using coupling gel, with the absence of ultrasound signal output in the sham group. Fundal height measurement and breast pain score were performed after each treatment. The primary outcome, uterine involution, was presented by measuring the fundal height of the uterus. The visual analogue scale (VAS) score, as a secondary outcome, was used to assess breast pain and determine the correlation between breast pain and fundal height as the outcome simultaneously. All participants were randomly assigned to either the LIFU group (n = 88) or sham group (n = 88), with seven individuals not completing the treatment. Overall, a statistically significant difference was noted in the rate and index of fundus descent after each treatment. The rate and index of fundus descent showed greater significance following the second treatment (rate: 1.5 (1.0, 2.0) cm/d; index: 0.15 (0.1, 0.18), *P* < 0.001) and third treatment (rate: 1.67 (1.33, 2.0) cm/d; index: 0.26 (0.23, 0.3), *P* < 0.001) in the LIFU group. VAS scores, which were based on the continuous variables for the baseline, first, second, and third treatments in the LIFU group (2.0 (2.0, 3.0), 1.0 (0.0, 2.0), 0.0 (0.0, 1.0), and 0.0 (0.0, 0.0) points, respectively), and the sham group (2.0 (2.0, 2.0), 2.0 (1.0, 2.0), 2.0 (1.0, 3.0), and 3.0 (1.0, 3.0) points, respectively), showed a statistically significant difference between the two groups. Meanwhile, the discrepancies in VAS score classification variables between the two groups were statistically significant. After the third treatment, a notable correlation was observed between the VAS score decrease and fundus descent rate; the more the VAS score decreased, the faster was the fundal decline rate in the LIFU group. LIFU therapy is safe and effective, contributing to the acceleration of uterine involution and the relief of postpartum breast pain.

*Trial ID* The study has registered in the Chinese Clinical Trial Registry (ChiCTR2100049586) at 05/08/2021.

## Introduction

After childbirth, women may encounter various postpartum issues, including breast pain and uterine involution, presenting prevalent clinical challenges that pose difficulties in effective resolution. With the rise in the average maternal age and an increasing number of older adult parturients, there has been a notable surge in the incidence of poor uterine involution^1^. Weakness in postpartum uterine contractions stands as a primary cause of postpartum haemorrhage, constituting a serious threat to the mother’ life. Postpartum breast pain predominantly arises from the rapid increase in postpartum milk production and subsequent insufficient milk discharge. The pain not only affects the ability to breastfeed but can also prematurely terminate breastfeeding in the event of serious complications. Multiple studies have demonstrated that problems with postpartum breastfeeding can impact uterine contractions and further influence uterine regeneration^2–4^.

Traditional clinical methods for achieving therapeutic effects in uterine involution exhibit difficulties and limitations. For example, while oxytocin is commonly used as a clinical treatment agent, its efficacy is hindered by reduced receptor sensitivity and individual differences^5^. Manipulation massage is one of the methods to alleviate breast pain; however, controlling the strength of manipulation is critical, as excessive force may lead to tissue damage^6,7^. Ultrasound has been widely employed in the treatment of musculoskeletal diseases, and its effectiveness and safety have been recognized as an acoustic mechanical wave. Low-intensity focused ultrasound (LIFU) can focus sound waves to achieve therapeutic energy release in deep tissues. It has been applied to several diseases, encompassing neurological diseases and tumours^8,9^. Despite studies have investigated the application of ultrasound in postpartum uterine involution and lactation following caesarean section and vaginal birth, particularly in China^10–13^, a dearth of clinical data from randomised controlled studies remains evident.

Therefore, a multicentre, single-blind, randomised clinical trial was conducted to evaluate the efficacy and safety of LIFU in treating postpartum uterine contractions and breast pain, thereby providing a new and effective treatment approach addressing prevalent postpartum complications.

## Methods

### Participants

This multicentre, randomised, sham-controlled, blinded clinical trial was conducted in the Obstetrics and Gynaecology Department at three medical centres in China from December 2018 to October 2019. Pregnant women at or beyond 37 weeks of gestation, without serious complications during prenatal check-ups, engaging in breastfeeding, and adhering to postnatal exercise as prescribed by their physicians, were included in this study. The following were the exclusion criteria: placenta previa; intrahepatic cholestasis of pregnancy; triplets, twins, fetal macrosomia; polyhydramnios; any history or signs of placental abruption; severe preeclampsia; and anticoagulant treatments, such as those for thrombocytopenia.

**Figure 1:**
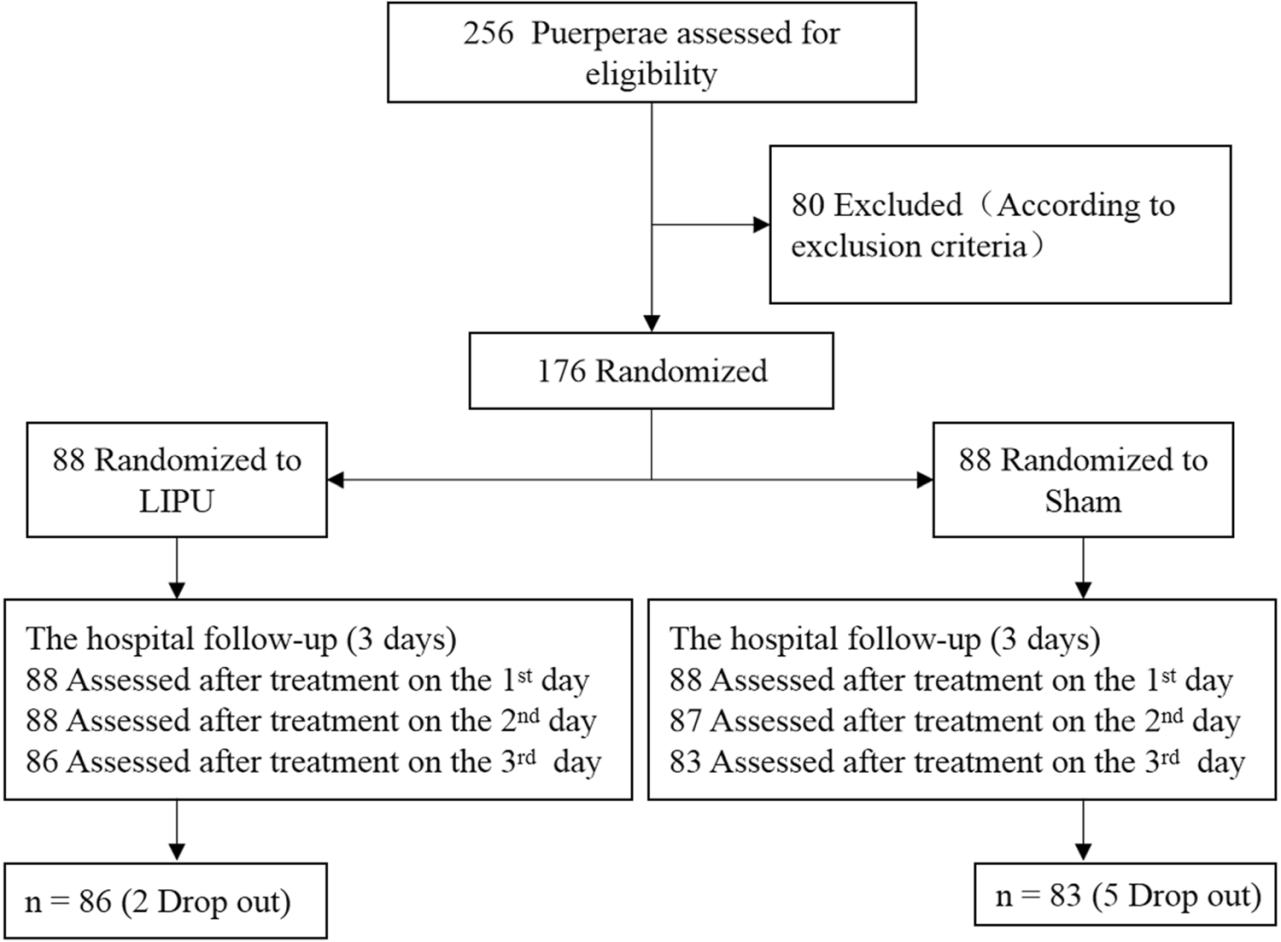
Flow of the puerpera through the study of LIFU.

A total of 256 participants were initially eligible, out of whom 176 were enrolled across the three centres and randomised into either the LIFU or sham group. Seven participants (two in the LIFU group and five in the sham group) did not comply with the study protocol (Fig. 1). Additionally, written informed consent was obtained from all participants before enrollment. The Clinical Trial Ethics was approved by Ethics Committee of West China Second Hospital of Sichuan University on May 11, 2018 (approval No. Q2018003). All methods were performed in accordance with the Ethics regulations, and the manuscripts reporting the clinical trial results conformed to CONSORT 2010 guidelines.

### Study design

Participants were divided into different groups stratified by the medical centre utilizing randomization methods based on a random sequence generated by a computer and independent data manager. Each random number was sealed in an envelope to conceal the sequence. The subjects were aware of the treatment assigned to them, whereas the surgeons, other clinicians, data collectors, investigators, data analysts, and industry sponsors were blinded to treatment allocation. The doctors enrolled subjects, and one doctor assigned participants to the respective groups. The participants in the LIFU group received a Model-TY-200A ultrasound therapeutic device (Sichuan Taiyou Technology Co., Ltd., China) for treatment targeting the uterus and breast. The following were the parameters used: an operating frequency of 0.7–0.9 MHz, a focus area of 0.07–0.085 cm^2^, a spatial peak time average sound intensity of < 3 W/cm^2^, and a pulse duty cycle of 25%–100%. The deactivated device (identical in appearance and operating function) was provided to the sham-controlled groups. The participants were instructed to empty their bladders and lie in a supine position. After fully exposing the treatment site, the therapist palpated the abdomen to locate and mark the fundus. Following the application of an appropriate coupling agent, the ultrasound head was gently aligned with the fundal position and moved at a constant speed (1–2 cm/s) around the fundus for treatment. Prior to breast treatment, the therapist identified and marked the pain site. The entire breast was treated at a consistent speed, circumventing the nipple and areola, and subsequently circling the pain site during the treatment. Generally, ultrasound therapy elicits no sensation or a mild warmth. Increasing the dosage of the coupling agent and altering the movement speed are required, when the participants develop obvious pain or an unbearable burning sensation. The treatment method for the sham operation group was the same as that of the LIFU group in therapy frequency and duration, but without any increase in energy. The first treatment commenced 6 h and 24 h after a vaginal birth and a caesarean section, respectively. The LIFU group received daily treatment for 3 consecutive days, with 30 min for each uterine treatment, and 20 min for each breast treatment (10 min on one side). Furthermore, all participants underwent routine treatment and care.

The fundal height, determined as the distance between the fundus and the midpoint of the superior margin of the pubic symphysis using a soft ruler, was measured by the same operator at each centre. Visual analogue scale (VAS) was utilized to assess breast pain, employing scorecards ranging from 0 to 10 for participants to select corresponding values according to their pain experience (0 indicating no pain and 10 indicating severe pain). Data on the fundal height and pain assessment scores were collected after each treatment.

### Evaluation criteria for efficacy

To evaluate uterine contraction, fundal decline served as the main outcome index, reflecting the condition of uterine involution. The significant difference in the rate (baseline height minus treatment height divided by the number of days) and index of fundus descent (baseline height minus treatment height divided by baseline height) between the LIFU and sham groups indicated treatment efficacy. The VAS score was employed as the secondary outcome to simultaneously assess breast pain; a notable disparity between the two groups denoted treatment efficacy. Moreover, correlations were investigated between the reduction of VAS score and the rate of fundus descent following the third treatment.

Safety evaluation included observing adverse events during the clinical trial, such as skin damage, allergies, and breast or abdominal pain.

### Statistical analyses

Descriptive statistics with 176 samples were calculated for demographic variables, including age and the number of pregnancies. Sample sizes were 176,175,169 for the first, second and third treatments respectively. The distribution characteristics and variable comparisons in the sham and LIFU groups were analyzed for statistical differences. The mean values (standard deviations) of continuous variables were used to represent the distribution of variables. The independent sample t-test was applied if the statistical test was normally distributed, and the Kolmogorov–Smirnov test was used to assess whether it followed the normal distribution; otherwise, the Mann–Whitney test was employed. The frequency of rank variables was statistically described, and the chi-square test was used to analyze the discrepancies between the two groups. The f-test was utilized to explore the significant correlation between breast pain and the rate of fundus descent. All analyses were performed using R 3.6.1 statistical software.

### Sample size

The PASS.15 software was used to calculate the sample size, based on a study in which the duration of lochia termination was 30.90 ± 9.15 days for patients receiving ultrasound therapy and 37.00 ± 11.21 days for those receiving deactivated device^14^. Two-sample t-tests were conducted to calculate the sample size, utilizing the mean and standard deviation to establish the effect size. With a two-sided alpha level of 0.05 and a statistical power of 90%, a calculation of 64 cases in each group was determined. In consideration of a 28-patient attrition rate (with a statistically acceptable 20% dropout and 8% other termination probability), an estimated sample of 176 patients would provide approximately 90% power to identify the between-group differences.

### Ethical statement

The Clinical Trial Ethics was approved by Ethics Committee of West China Second University Hospital, Sichuan University on May 11, 2018 (approval No. Q2018003).

## Results

### Baseline characteristics

The baseline data of 176 participants comprised age, history of caesarean section, number of pregnancies and births, fundal height, breast pain assessment, as well as routine blood investigations including haemoglobin, haematocrit, red blood cell count, white blood cell count, platelet count, and activated partial thromboplastin time. Analysis revealed well-balanced baseline characteristics among participants in both groups (Table 1).

**Table 1:** Baseline puerperal characteristics among women receiving LIFU with Sham.

Covariate	ALL (n = 176)	Sham (n = 88)	LIFU (n = 88)	*P*
Age (years)	31.28(4.47)	31.09(4.51)	31.47(4.44)	0.579
Caesarean section no. (%)	125 (72.25%)	61 (70.93%)	64 (73.56%)	0.828
HGB (g/L)	123.19(12.54)	123.4 (12.5)	122.97(12.65)	0.815
HCT (%)	28.07(16.3)	28.13(16.39)	28.02(16.29)	0.965
RBC (10^12^/L)	4.06(0.39)	4.09(0.41)	4.04(0.38)	0.406
WBC (10^9^/L)	9.86(6.65)	10.26(8.92)	9.46(2.99)	0.424
PLT (10^9^/L)	184.44(59.98)	183.44(60.16)	185.44(60.12)	0.826
APTT (sec)	26.74(2.46)	26.78(2.23)	26.71(2.69)	0.848
Number of pregnancies				0.338
1, no. (%)	59 (33.52)	26 (29.54)	33 (37.5)	
≥ 2, no. (%)	117(66.48)	62(70.46)	55(62.5)	
Number of births				0.879
1, no. (%)	98 (55.68)	48 (54.54)	50 (57)	
≥ 2, no. (%)	78(44.32)	40(45.46)	38 (43)	
Breast pain (VAS score)				0.141
0, no. (%)	4 (2.27)	1 (1.14)	3 (3.41)	
1, no. (%)	29 (16.48)	14 (15.91)	15 (17.05)	
2, no. (%)	90 (51.14)	52 (59.09)	38 (43.18)	
3, no. (%)	43 (24.43)	19 (21.59)	24 (27.27)	
4, no. (%)	8 (4.55)	1 (1.14)	7 (7.96)	
5, no. (%)	2 (1.14)	1 (1.14)	1 (1.14)	
Fundal height before treatment (cm)	19.41(2.64)	19.49(2.39)	19.34(2.88)	0.712

**HGB:** haemoglobin; **HCT:** haematocrit; **RBC:** red blood cell; **WBC:** white blood cell; **PLT:** platelet; **APTT:** activated partial thromboplastin time.

### Evaluation of the uterine fundus descent

The rate of fundus descent (calculated as the difference between baseline height and treatment height divided by the number of days) in the two groups after each treatment was analyzed by means of continuous variables. After the initial, second, and third rounds of treatment, the rate of fundus descent in the LIFU group escalated to 0.0(0.0, 1.0), 1.5(1.0, 2.0), and 1.67 (1.33, 2.0) cm/d, respectively. In contrast, the sham group exhibited a rise to 0.0 (0.0, 0.0), 1.0 (0.5, 1.25), and 1.0 (0.67, 1.0) cm/d over the same periods. Compared with the sham group, the rate of fundal descent in the LIFU group was faster, demonstrating a statistically significant difference after each treatment (*p* < 0.05). Moreover, the index of fundus descent (computed as the difference between baseline height and treatment height divided by baseline height) corroborated these findings; the descent index of the LIFU group was bigger than that of the sham group, with a significant statistical difference (*p* < 0.05) (Table 2).

**Table 2:** Evaluation of the uterine fundus descent M(*P*_25_,*P*_75_).

Rate of fundus descent (cm/d)	ALL	Sham	LIFU	H	*P*
First treatment	0.0(0.0, 1.0) (n = 176)	0.0(0.0, 0.0) (n = 88)	0.0(0.0, 1.0) (n = 88)	3230.5	0.021
Second treatment	1.0(0.5, 1.5) (n = 175)	1.0(0.5, 1.25) (n = 87)	1.5(1.0, 2.0) (n = 88)	2630	< 0.001
Third treatment	1.33(1.0, 1.67) (n = 169)	1.0(0.67, 1.0) (n = 83)	1.67(1.33, 2.0) (n = 86)	894	< 0.001

Index of fundus descent	ALL	Sham	LIFU	H	*P*
First treatment	0.0(0.0, 0.05) (n = 176)	0.0(0.0, 0.0) (n = 88)	0.0(0.0, 0.05) (n = 88)	3243	0.023
Second treatment	0.11(0.05, 0.17)(n = 175)	0.09(0.05, 0.12) (n = 87)	0.15(0.1, 0.18) (n = 88)	2448.5	< 0.001
Third treatment	0.2(0.14, 0.27) (n = 169)	0.15(0.11, 0.17) (n = 83)	0.26(0.23, 0.3) (n = 86)	505.5	< 0.001

### Evaluation of the postpartum breast pain

Continuous and discrete VAS scores were used separately for analysis. The numerical variables between the two groups provided insights into the overall situation, and the count variables categorized by the scores offered a more explicit view of the changes in the proportions of each grade before and after the intervention. The mean VAS score for each measurement showed a substantial statistical variance between the two groups (*p* < 0.05). Compared with the baseline, the VAS score at the initial treatment decreased to 1.0 (0.0, 2.0) and 2.0 (2.0, 3.0) in the LIFU and sham groups, respectively. Additionally, in the LIFU group, the VAS score dropped severally to 0.0 (0.0, 1.0) and 0.0 (0.0, 0.0) after the second and third treatments; while in the sham group, the VAS scores were 2.0 (1.0, 3.0) and 3.0 (1.0, 3.0) following the second and third treatments, correspondingly. In the sham group, the VAS score was higher than that at baseline after the third treatment (Table 3).

**Table 3:** VAS score based on the continuous variable for each measurement M(*P*_25_,*P*_75_).

Variable	ALL	Sham	LIFU	H	*P*
Baseline	2.0(2.0, 3.0) (n = 176)	2.0(2.0, 2.0) (n = 88)	2.0(2.0, 3.0) (n = 88)	3535.5	0.14
First treatment	2.0(0.0, 2.0) (n = 176)	2.0(1.0, 2.0) (n = 88)	1.0(0.0, 2.0) (n = 88)	2945	0.002
Second treatment	1.0(0.0, 2.0) (n = 175)	2.0(1.0, 3.0) (n = 87)	0.0(0.0, 1.0) (n = 88)	1245.5	< 0.001
Third treatment	1.0(0.0, 3.0) (n = 169)	3.0(1.0, 3.0) (n = 83)	0.0(0.0, 0.0) (n = 86)	503.5	< 0.001

According to the scoring results, the VAS score was divided into 0, 1, 2, 3, 4, and 5 categories, and the frequency distribution was used to make statistics for each measurement. After the first, second, and third treatments, the number of participants reporting no pain (score 0) increased to 41 (46.59%), 54 (61.36%), and 70 (81.4%), respectively; however, the numbers were 16 (18.18%), 7 (8.05%), and 4 (4.82%) in the sham group during the same periods. A statistically significant difference was noted between the two groups, with *P* < 0.05 as per the chi-square test (Table 4).

**Table 4:** VAS score based on the categorical variable for each measurement n(%).

Variable	ALL (n = 176)	Sham (n = 88)	LIFU (n = 88)	χ^2^	*P*
Baseline				8.29	0.141
0	4 (2.27%)	1 (1.14%)	3 (3.41%)		
1	29 (16.48%)	14 (15.91%)	15 (17.05%)		
2	90 (51.14%)	52 (59.09%)	38 (43.18%)		
3	43 (24.43%)	19 (21.59%)	24 (27.27%)		
4	8 (4.55%)	1 (1.14%)	7 (7.96%)		
5	2 (1.14%)	1 (1.14%)	1 (1.14%)		
First treatment				18.77	0.002
0	57 (32.39%)	16 (18.18%)	41 (46.59%)		
1	29 (16.48%)	20 (22.73%)	9 (10.23%)		
2	58 (32.96%)	34 (38.64%)	24 (27.27%)		
3	28 (15.91%)	16 (18.18%)	12 (13.64%)		
4	3 (1.71%)	2 (2.27%)	1 (1.14%)		
5	1 (0.57%)	0 (0.0%)	1 (1.14%)		

Variable	ALL (n = 175)	Sham (n = 87)	LIFU (n = 88)	χ^2^	*P*
Second treatment				66.01	< 0.001
0	61 (34.86%)	7 (8.05%)	54 (61.36%)		
1	41 (23.43%)	22 (25.29%)	19 (21.59%)		
2	48 (27.43%)	34 (39.08%)	14 (15.91%)		
3	16 (9.14%)	15 (17.24%)	1 (1.14%)		
4	8 (4.57%)	8 (9.2%)	0 (0.0%)		
5	1 (0.57%)	1 (1.15%)	0 (0.0%)		

Variable	ALL (n = 169)	Sham (n = 83)	LIFU (n = 86)	χ^2^	*P*
Third treatment				108.27	< 0.001
0	74 (43.79%)	4 (4.82%)	70 (81.4%)		
1	30 (17.75%)	19 (22.89%)	11 (12.79%)		
2	19 (11.24%)	16 (19.28%)	3 (3.49%)		
3	25 (14.79%)	24 (28.92%)	1 (1.16%)		
4	17 (10.06%)	16 (19.28%)	1 (1.16%)		
5	4 (2.37%)	4 (4.82%)	0 (0.0%)		

### Correlation between breast pain and uterine fundus in the two groups

**Figure 2:**
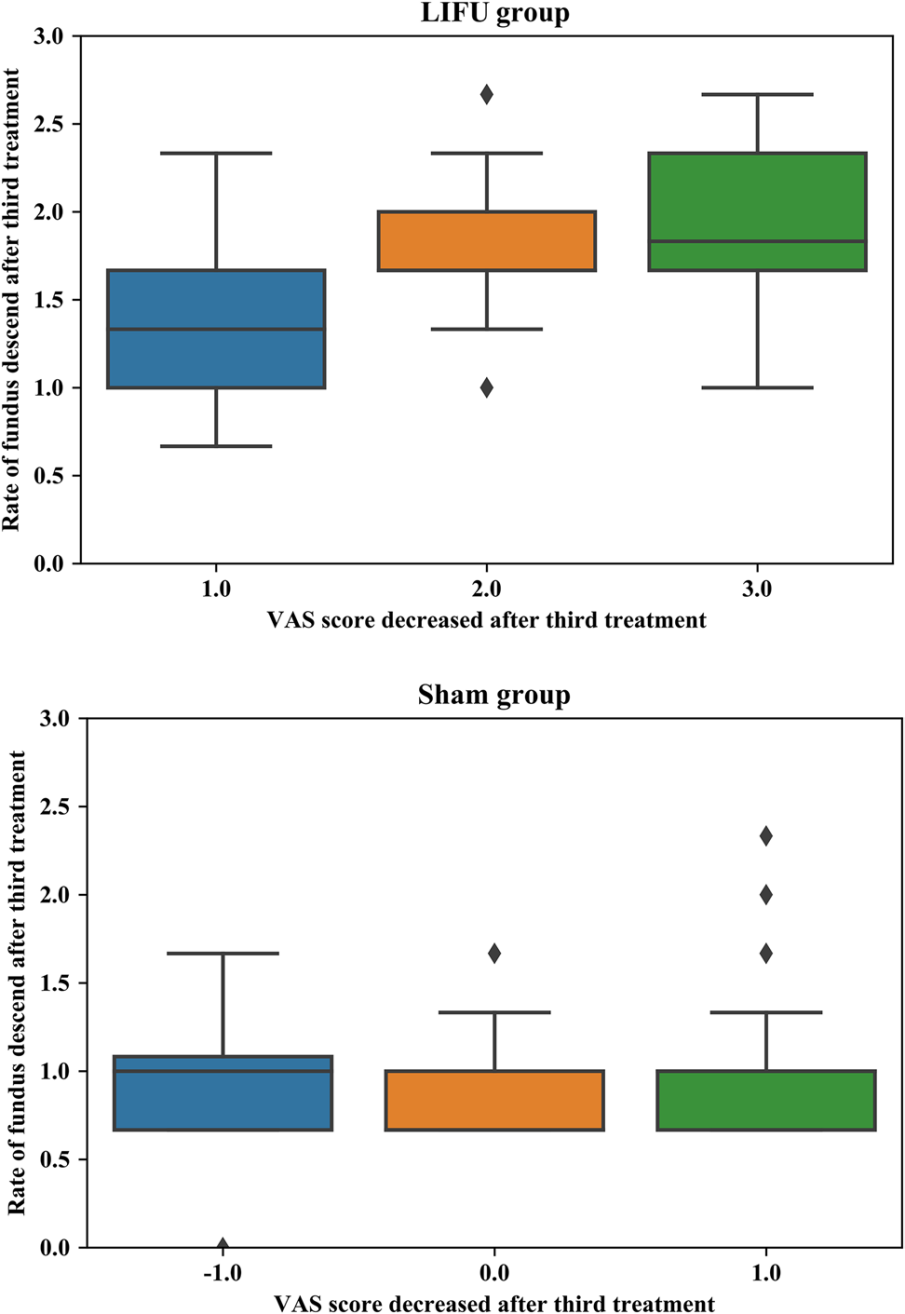
Correlation between the VAS score decrease and rate of fundus descent after the third treatment.

In the sham group, the VAS scores of the participants diminished from the baseline to the third treatment) and were mainly concentrated in the − 1, 0, and 1 categories, whereas the VAS scores of those in the LIFU group were mainly in the 1, 2, and 3 categories. The corresponding rates of fundus descent were 0.97 cm/d, 0.9 cm/d, and 1.05 cm/d in the sham group and 1.4 cm/d, 1.71 cm/d, and 1.93 cm/d in the LIFU group, respectively. The decrease in VAS scores was significantly correlated with the third fundus descent rate in both the LIFU (F statistic = 5.27, *P* = 0.023) and sham groups (F statistic = 87.687, *P* < 0.001). In the LIFU group, as the VAS scores decreased, the fundus descent rate increased. However, in the Sham group, despite the reduction in VAS scores, there was no observed change in the rate of fundal descent (Fig. 2).

### Safety evaluation

Throughout the study, none of the participants developed any adverse reactions, including abdominal pain, skin allergy, or skin injury.

## Discussion

Uterine involution and breast pain are the most common postpartum clinical concerns. Furthermore, postpartum uterine contractions can be elicited not only by oxytocin administration but also by mechanical stimulation methods including manual compression. Additionally, manipulation massage of mechanical stimulation, which aids in dredge the breast ducts to facilitate milk discharge, is performed to relieve postpartum breast pain. While, the manipulation process is painful and unbearable, clinical manipulative interventions also indicate that mechanical stimulation might be an effective method. Therefore, there is a necessity for painless, highly penetrating, and non-invasive mechanical stimulation therapies. As a mechanical wave, ultrasonic wave is penetrating and spreads in a straight line with a good target property. The LIFU used in this study focuses ultrasonic beams to release therapeutic energy into deep tissues, thereby penetrating uterine tissues and the painful breast site.

Our preliminary results showed that the participants receiving LIFU treatment exhibited a swifter decline rate and a higher decline index compared to those undergoing sham treatment. This suggested that LIFU was effective in the treatment of uterine contractions. The biological effects of LIFU’s mechanical stimulation, combined with its physical advantages of high penetration and focusability, render the application of LIFU for uterine involution comfortable and painless^15,16^. The uterine smooth muscle tissue is incredibly sensitive to mechanical stimulation at specific frequencies, resulting in contractions. Low-intensity ultrasound may promote uterine smooth muscle contractions by modulating the cell-membrane permeability to Ca^2+^, thus facilitating the influx of extracellular fluid Ca^2+^ and/or the release of intracellularly stored Ca^2+^^17^. Notably, a slight change was observed in the rate of fundus descent. After the first treatment, there was almost no decline in the rate of fundus descent in the sham group (on postpartum day 1), whereas the rate in the LIFU group was also slower compared to the subsequent treatments. This might be attributed to the uterine state on the first day after birth. The uterine contractions of a larger proportion of participants who undergone cesarean delivery, affected by surgery and uterine hematosis, were slower during the initial postpartum day^18^. Nonetheless, following the first treatment, the LIFU group also noticeably enhanced uterine contraction with statistical significance.

Moreover, this clinical trial was the first to apply LIFU for postpartum breast pain, demonstrating a significant therapeutic impact, with 81.4% of the participants experiencing no pain following the three treatments (VAS score = 0). LIFU emerged as a promising physical factor analgesic method for managing postpartum breast pain. The outcomes manifested that most participants did not develop recurrent pain after three consecutive treatments, indicating that LIFU not only exerted an analgesic effect on the breast but might also facilitate breast dredging and promote circulation to alleviate pain^19–22^.

Numerous studies have confirmed that postpartum breastfeeding stimulated the mammary gland, which caused uterine contractions and promoted uterine involution^2–4^. One study revealed that, at 3 months postpartum, women maintaining an daily breastfeeding rate of 80% or higher exhibited shorter uterine lengths compared to those with the rate of 20% or less^23^. Furthermore, our current investigation substantiated the correlation between the mammary gland and uterine contraction. Through an analysis of breast pain and the rate of fundus descent, our findings underscored a correlation between these factors. Importantly, our study demonstrated that participants experiencing significant relief from breast pain following LIFU treatment exhibited an accelerated rate of uterine descent. Postpartum breast pain primarily arises from breast swelling and difficulty in milk discharge. LIFU treatment can unblock breast ducts, ensuring timely and effective milk emptying and facilitating smooth milk secretion. This stimulation can promote oxytocin secretion, reduce postpartum bleeding, and further improve uterine contraction, thus possibly providing a clinical rationale for the results of our study. Additionally, our findings suggested that LIFU treatment not only directly promoted uterine retraction but also influenced uterine recovery in managing breast pain. Moreover, our results also indicated that LIFU could be simultaneously applied to both the breast and uterus in clinical practice to better solve postpartum issues.

According to the study, LIFU proved to be a safe, painless and effective physical therapy. Its capabilities in high penetration and focusing allowed for reaching the depths of the uterus and mammary gland with efficacy. Additionally, the treatment process also proved comfortable and painless. Notably, this marked the pioneering use of ultrasound in the treatment of postpartum breast pain.

This study highlighted LIFU's significant effectiveness in accelerating uterine retraction therapy; however, further investigations involving long-term follow-ups and larger sample sizes were imperative. While, LIFU displayed notable efficacy in relieving breast pain, additional evaluation methods, such as ultrasound imaging, are warranted to corroborate its efficacy. Future endeavors should include well-designed randomised controlled trials with larger sample sizes to validate these findings.

## Conclusion

The findings of this study revealed that LIFU was a safe and effective method in stimulating postpartum uterine contractions and relieving postpartum breast pain. The results verified the correlation between breast pain and uterine contraction, suggesting that the simultaneous clinical application of LIFU intervention on the breast and uterus, which was conducive to postpartum rehabilitation. Notably, LIFU proved painless and devoid of side effects. showcasing its potential for widespread clinical adoption in treating uterine involution and breast pain. Further exploration into LIFU's varied applications within the realm of postpartum rehabilitation warrants additional research.

## Data Availability

The datasets generated and/or analysed during the current study are available in the [Resman Research Manager] repository, [http://www.medresman.org.cn/pub/cn/proj/projectshshow.aspx?proj=3256].
